# Acoustical Direction Finding with Time-Modulated Arrays

**DOI:** 10.3390/s16122107

**Published:** 2016-12-11

**Authors:** Ben Clark, James A. Flint

**Affiliations:** Wolfson School of Mechanical, Electrical and Manufacturing Engineering, Loughborough University, Leicestershire LE11 3TU, UK; james.flint@ieee.org

**Keywords:** time-modulation, linear array, MEMS, direction of arrival

## Abstract

Time-Modulated Linear Arrays (TMLAs) offer useful efficiency savings over conventional phased arrays when applied in parameter estimation applications. The present paper considers the application of TMLAs to acoustic systems and proposes an algorithm for efficiently deriving the arrival angle of a signal. The proposed technique is applied in the frequency domain, where the signal and harmonic content is captured. Using a weighted average method on harmonic amplitudes and their respective main beam angles, it is possible to determine an estimate for the signal’s direction of arrival. The method is demonstrated and evaluated using results from both numerical and practical implementations and performance data is provided. The use of Micro-Electromechanical Systems (MEMS) sensors allows time-modulation techniques to be applied at ultrasonic frequencies. Theoretical predictions for an array of five isotropic elements with half-wavelength spacing and 1000 data samples suggest an accuracy of $\pm 1^{\circ }$ within an angular range of approximately $\pm 50^{\circ }$. In experiments of a 40 kHz five-element microphone array, a Direction of Arrival (DoA) estimation within $\pm 2.5^{\circ }$ of the target signal is readily achieved inside a $\pm 45^{\circ }$ range using a single switched input stage and a simple hardware setup.

## 1. Introduction

There are numerous situations where the range and origin of a transmitting source need to be determined. Some of the most common methods that achieve this use a receiver that electronically or physically scans space to find the Direction of Arrival (DoA). Electronic systems usually incorporate an array of sensors [1,2,3].

Once the input data is gathered it can be post-processed for DoA applications by established techniques such as the "MUltiple SIgnal Classification" (MUSIC) [4] and "Estimation of Signal Parameters by Rotational Invariance Techniques" (ESPRIT) [5] algorithms. These algorithms make use of sub-space methods which involve a significant amount of computation to produce an accurate direction estimation for an incoming signal; furthermore, the data of each element in the array usually needs to be recorded concurrently. Systems with the specifications to perform these calculations can become expensive and therefore there is a need to reduce complexity for smaller, low-cost devices. One such device may take the form of a fully integrated Micro-Electromechanical Systems (MEMS) sensor with multiple sensor elements, and a low-cost processing element.

Ultrasonic systems in air for applications such as robotic or vehicle guidance [6,7] may also use an array of transducers, and use techniques such as Time Difference of Arrival (TDoA) [8] or phase steering [9] to obtain a DoA estimation. For TDoA techniques, the use of cross-correlation requires each sensor to be sampled; for phase steered arrays, each element’s phase needs to be controlled to produce a beam in a particular direction, which is usually swept across a range of directions. The present paper considers electrically scanned systems operating in the receive mode only which utilise Time-modulated Linear Arrays (TMLAs). TMLAs function by sampling the array in a switched fashion rather than by means of phase steering [10] and benefit from only requiring a single analogue input stage with the sensors being switched on periodically to drive that input. The switching waveforms and frequencies can be varied in order to change the properties of the array.

**Figure 1:**
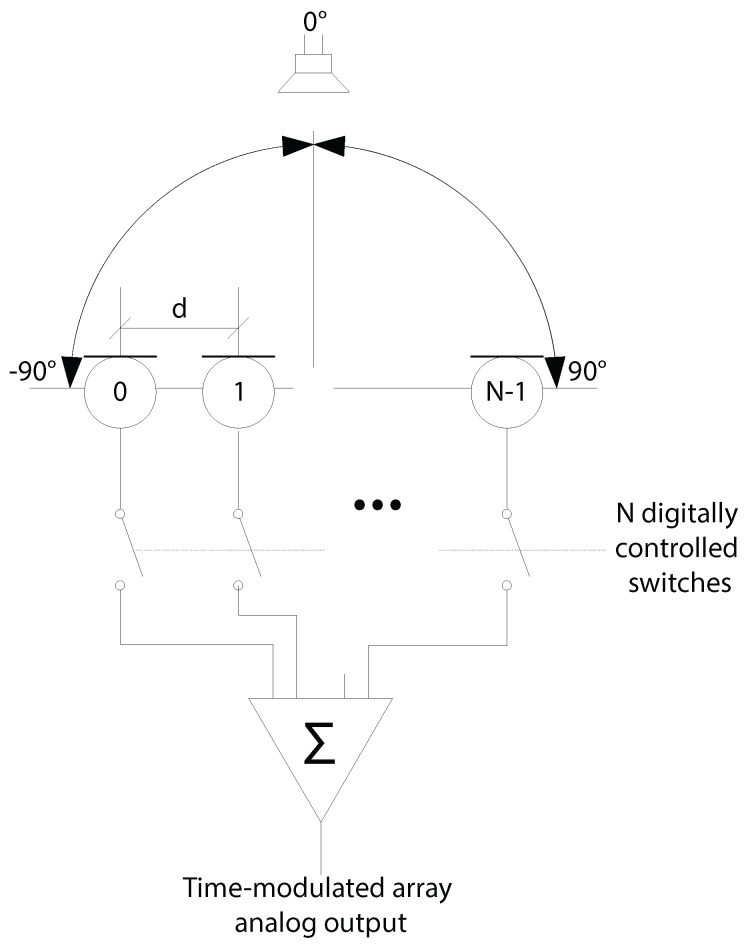
Typical schematic of an *N* element time-modulated array with *d* spacing.

The use of time modulation was originally conceived by Shanks and Bickmore [10] as a method of increasing the capabilities of transducer arrays. In a time-modulated array (Figure 1), elements can be periodically switched on and off. Due to the nature of the switching, power in the frequency spectrum is distributed amongst the signal and harmonic components related to the switching frequencies. This effect can be controlled by changing the switching waveform [11].

Time modulated arrays have received increased interest recently in direction-finding applications due to their inherent benefits, and although the MUSIC algorithm has been adapted for use with time-modulated arrays [12,13] other researchers have found that the study of the harmonic components is all that is required for DoA estimation and as a result fast algorithms that have low computational complexity have been developed [14,15].

He [15] has demonstrated that it is possible to extract an estimation for a signal’s DoA using a two element time-modulated array by performing a two-point Discrete Fourier Transform (DFT). Whilst this method can significantly reduce the complexity of the algorithms, it can also be prone to errors due to multipath reflections and noise, especially in situations where a small number of samples are captured.

The present paper suggests an alternative computationally efficient method using TMLAs to estimate the DoA and demonstrates the method’s effectiveness when used in a five-element broadband microphone array to locate a 40 kHz signal in air. The use of this method with larger numbers of sensors shows that DoA estimations can be accurately produced even in the presence of noise and multi-path reflections.

## 2. Theory

### 2.1. Background

The normalized array factor of an *N* element, TMLA when receiving a signal from direction *θ* can be written as [16]

(1) $$AF\left(\theta ,t\right)=\frac{1}{N}\sum_{n=0}^{N-1}U_{n}\left(t\right)e^{jknd\;\sin\theta }$$

where *k* is the wavenumber (rad/m), *n* is the element number, *d* is the spacing between elements in metres, and $U_{n}\left(t\right)$ is a time-switching function that turns each element either off or on in time defined by,

(2) $$U_{n}\left(t\right)=\left\{\begin{array}{cc} 1, & \tau_{n,on}\leq t<\tau_{n,off}\\ 0, & \mathrm{otherwise} \end{array}\right.$$

where $\tau_{n,on}$ and $\tau_{n,off}$ are the switch on and off times respectively for the *n*th element in the array.

If each element is switched on then off uniformly in sequence, $U_{n}\left(t\right)$ becomes a periodic function and can be formulated as an infinite sum of *m* Fourier components each with coefficient $c_{m,n}$ [17]

(3) $$U_{n}\left(t\right)=\sum_{m=-\infty }^{\infty }c_{m,n}e^{-j2\pi mf_{s}t}$$

where the coefficients can be calculated as [11]

(4) $$\begin{array}{cc} c_{m,n} & =\frac{1}{T_{s}}\int_{0}^{T_{s}}U_{n}\left(t\right)e^{-jm2\pi f_{0}t}dt\\ & =\frac{\sin\frac{\pi m}{N}}{\pi m}e^{\frac{-j2\pi mn}{N}} \end{array}$$

The signal power is distributed among the harmonics of the switching pattern frequency $f_{s}=1/T_{s}$, centred on a main frequency $f_{0}$. Using the above equations, the array factor for a particular harmonic *m* can be calculated as,

(5) $$AF\left(m,\theta ,t\right)=\frac{1}{N}\frac{\sin\frac{\pi m}{N}}{\pi m}e^{-j2\pi (f_{0}+mf_{s})t}\sum_{n=0}^{N-1}e^{\frac{-2j\pi mn}{N}}e^{jknd\;\sin\theta }$$

and if *R* is defined as the ratio of element spacing to signal wavelength *λ*,

(6) $$R=\frac{d}{\lambda }=\frac{kd}{2\pi }$$

then the normalized array factor for each harmonic can be written as

(7) $$AF\left(m,\theta ,t\right)=\mathrm{sinc}\left(\frac{m}{N}\right)e^{-j2\pi (f_{0}+mf_{s})t}\sum_{n=0}^{N-1}e^{2j\pi nR(\sin\theta -\frac{m}{RN})}$$

**Figure 2:**
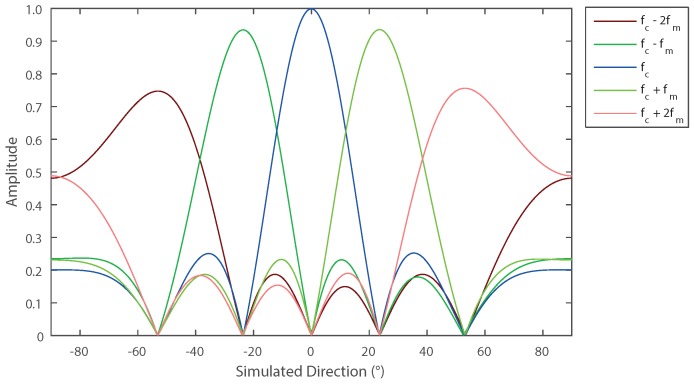
Simulated Array Factor of a five-element array, for five different harmonics when each element is switched on and off uniformly in sequence.

Figure 2 shows the normalized response of the dominant harmonics (i.e., $f_{c}\pm 2f_{m}$, $f_{c}\pm f_{m}$, $f_{c}$) in a five-element array with each harmonic having a maximum amplitude at a different angle (i.e., $\pm 53.1^{\circ }$, $\pm 23.6^{\circ }$, $0^{\circ }$). From Equation (7), it can be shown that the direction in which the maximum response occurs for each harmonic can be calculated as:

(8) $$\theta_{m}=\sin^{-1}\frac{m}{RN}$$

It can also be observed that in the case of a uniformly switched array (i.e., ($\tau_{n,off}-\tau_{n,on})\times N=T_{s}$, and each element is switched on in sequence), the point at which each harmonic’s maximum amplitude occurs also marks the point where all other harmonics are at a minimum. The signal power associated with each frequency can be easily identified using a Discrete Fourier Transform (DFT). In practical terms this means that by analytical study of the amplitudes of each harmonic’s DFT bin, a value for a given received signal’s DoA can be derived.

Array factors associated with each harmonic depend on the value of *R* as defined in Equation (6). As *R* becomes smaller, the main beam angles of each harmonic as calculated in Equation (8) will be more widely spaced. If *R* is greater than 0.5 (half-wavelength spacing), harmonics will obtain grating lobes due to the effect of spatial aliasing; in these cases, there are multiple angles in which harmonics become responsive, and the problem of direction-finding becomes more numerically complex. Throughout this paper, *R* is chosen to be 0.5 to ensure that each of the harmonics has a single main beam within the range of $\pm 90^{\circ }$.

### 2.2. Proposed Method of Determining the DoA

The approach taken in this paper is realised by finding the associated harmonic frequencies relating to both the signal frequency and the array switching frequency. For example, an array with five elements (N=5) will distribute the power of an incoming signal into frequencies at $f_{c}-2f_{m}$, $f_{c}-f_{m}$, $f_{c}$, $f_{c}+f_{m}$ and $f_{c}+2f_{m}$. As shown in Equation (8), each of these harmonics has a known main beam angle $\theta_{m}$ (i.e., $-53.1^{\circ }$, $-23.6^{\circ }$, $0^{\circ }$, $23.6^{\circ }$ and $53.1^{\circ }$).

Consider a single frequency sinusoidal signal impinging on the array at an angle *θ* measured relative to the broadside direction. This signal will cause one or more frequency bins to become filled (as demonstrated by Figure 2) according to which harmonics are detected at the receiver. In the case where *θ* matches exactly a value of $\theta_{m}$, only one frequency is expected to be present in the output. It can then be assumed that if the value of the largest DFT bin is dominant, while the adjacent bins are small, then the signal angle is close to the main beam angle of that harmonic. Conversely, if the amplitudes of two adjacent bins are similar and also the largest measured, then the signal is approximately half way between the two main beams. The DoA can be estimated by looking at the average angle created by two beams, as weighted by the harmonic power level the array receives; estimation of the DoA can be expressed as:

(9) $$\theta_{est}=\frac{X_{\alpha }\theta_{\alpha }+X_{\alpha +1}\theta_{\alpha +1}}{X_{\alpha }+X_{\alpha +1}}$$

where $X_{\alpha }$ and $X_{\alpha +1}$ are the two largest DFT bins that are adjacent to each other, and $\theta_{\alpha }$ and $\theta_{\alpha +1}$ are the main beam angles associated with the corresponding harmonics.

The steps required to perform DoA estimation using the proposed method can be summarised as follows:

1. Collect a fixed number of samples from each microphone in turn (this can be repeated multiple times, however it is often adequate to do this just once). The harmonic frequency can be calculated as the reciprocal of the time taken to sample the set of all sensors.
2. Perform a DFT on the fundamental and expected harmonics of the switching frequency.
3. Select the DFT bins with the two largest amplitudes and identify their main beam angles.
4. Calculate a DoA using Equation (9)

**Figure 3:**
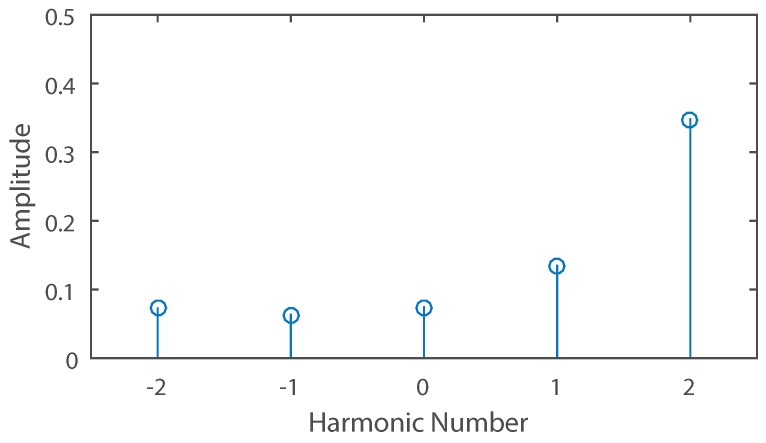
Power spectrum produced by a single sinusoidal source when sampled by a uniformly switched five-element time modulated linear array. The signal is impinging on the array at an angle of $45^{\circ }$.

For example, if an unknown signal source produces the frequency spectrum shown in Figure 3 when sampled with a five-element array, then the power levels of harmonics 1 and 2 are taken as $X_{\alpha }$ and $X_{\alpha +1}$ respectively and angles $\theta_{\alpha }$ and $\theta_{\alpha +1}$ are found from Equation (8) as $23.6^{\circ }$ and $53.1^{\circ }$. Using Equation (9), the estimated signal DoA is $44.9^{\circ }$.

#### 2.2.1. Error Reduction Technique

**Figure 4:**
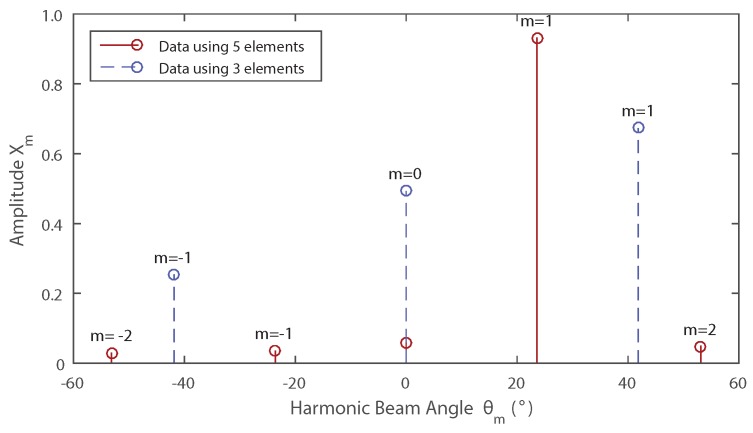
Power spectrum produced by a five-element array receiving a signal from a source at an angle of $25^{\circ }$. When the samples from the outer elements are removed, then the array produces a new spectrum with different characteristics (shown as the dotted lines in the figure).

Figure 4 shows the amplitudes of each harmonic plotted against the associated main beam angles. The source in this example is located at $25.0^{\circ }$ and the solid lines indicate the result for (N=5). In this situation, the choices of $X_{\alpha }$ and $X_{\alpha +1}$ are not clear. Harmonic 1 (m=1) is the dominant frequency, but the two adjacent harmonics (m=0, m=2) are very similar in amplitude; both make possible candidates for the selection of $X_{\alpha }$ or $X_{\alpha +1}$. Using the method described, harmonics 0 and 1 will produce an estimation of $22.0^{\circ }$ whereas using harmonics 1 and 2 will produce an estimation of $25.0^{\circ }$. Clearly, harmonic 2 is the correct value to use, however determining which of the two small harmonics can be challenging especially in the presence of noise. It can be concluded following this argument that the worst error case occurs when the incident angle of the signal is close to one of the harmonics’ main beam angles.

This issue can be mitigated by processing the sampled data in a different way. If a number of samples has been taken from each element in turn but not repeated, it is possible to remove the samples recorded by the outer elements so that data from an N−2 element array is obtained. This produces a different frequency spectrum due of the effect of removing elements, the modulation frequency becomes higher and the resultant harmonic beam directions change according to Equation (8). By selecting the number of elements used, errors caused by estimating the source direction using harmonic amplitudes close to the null regions can be avoided. Using the data provided, if harmonics 0 and 1 are taken as $X_{\alpha }$ and $X_{\alpha +1}$ in the three-element case then the method produces an estimation of $24.6^{\circ }$.

## 3. Numerical Results

**Figure 5:**
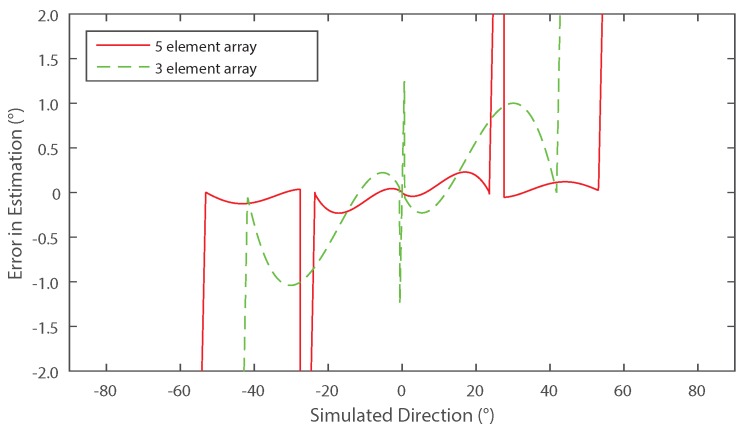
Simulated error in direction of arrival estimation of a linear array using five (solid) or three microphones (dashed).

In this section, results of a simulation developed in MATLAB R2015a are provided to illustrate the use of the proposed technique. A source of 40 kHz was assumed, impinging on a five-element array, where each element was separated by λ/2. A sampling rate of 500 kHz was chosen and 200 samples were taken from each simulated isotropic receiver in succession, meaning that the expected harmonics are separated by 500 Hz. The results are shown Figure 5 (solid line) which gives the accuracy of the DoA estimation as a function of source angle.

**Figure 6:**
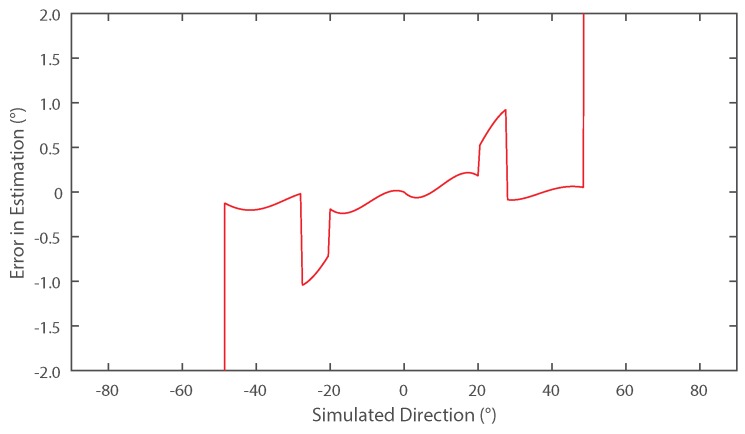
Simulated error in direction of arrival estimation when the best result is picked from a linear array using five or three of its elements.

The abrupt changes in estimation error observed at approximately $23^{\circ }$–$25^{\circ }$ for the five-element array in Figure 5 are due to the algorithm selecting an incorrect smaller harmonic close to the null regions as explained in Section 2.2.1. In spite of there being no noise in this simulation, effects such as under-sampling and numerical errors still give rise to errors close to the null. If now we consider the estimation error for N=3 (shown as the dotted line in Figure 5) and then the results are re-analysed using the same method with new values of harmonic data, the inaccuracies can be mitigated by taking a composite of the two traces. Since the value of *N* has changed, the null regions located using Equation (8) for N=3 and N=5 are non-coincident. It is known at which angles the inaccuracies will occur and hence by selecting the result of either N=3 or N=5, the best case accuracy overall can be achieved as shown in Figure 6.

**Figure 7:**
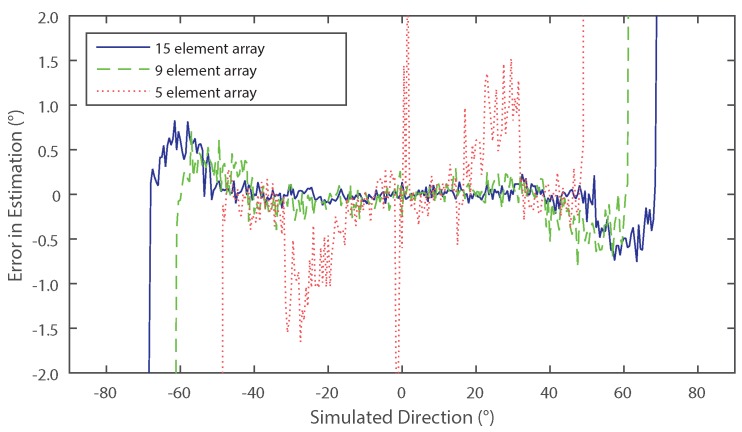
Simulated error in direction of arrival estimation with three different numbers of elements in the presence of noise. Each test had 1350 samples taken from an array with five (dotted), nine (dashed) or fifteen (solid) elements, where the source signal to noise ratio was 10 dB.

Multiple values of *N* were tested using a single source with additive white Gaussian distributed noise. The signal to noise ratio was fixed throughout all tests at 10 dB. Figure 7 shows that using a larger number of elements increases the accuracy of the method, as well as increasing the effective angular range of the system.

Using this method, it is also possible to detect multiple incoherent signals simultaneously if the frequency of the second signal and its harmonics generated by switching do not interfere with the DFT approximations performed on the first signal and its harmonics. In this case, the designer of the system must space the microphones at less than half a wavelength apart at the highest frequency to avoid spatial aliasing.

## 4. Experimental Results

**Figure 8:**
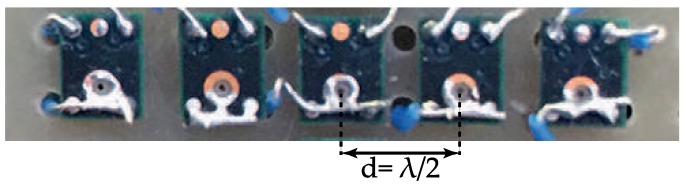
Setup of the experimental microphone array with an inter-element spacing (d) of approximately λ/2 where *λ* is the wavelength of a 40 kHz acoustic wave. Each microphone is a SPU0410LR5H-QB [18] Micro-Electromechanical Systems (MEMS) omnidirectional microphone.

A linear five-element microphone array using MEMS microphones was set up (Figure 8) with an inter-element spacing of 4.25 mm. This array spacing was chosen to be half the wavelength of a signal transmitted from a narrowband 40 kHz piezoelectric acoustic source. This source was a generic device of type “400ST160” having a bandwidth of approximately 2 kHz and a transmit pressure of approximately 110 dB Sound Pressure Level (SPL). It was driven from the 50Ω output of a function generator producing a 40 kHz sine wave. A National Instruments “myRIO” was used for the processing of the received signal. The Field-Programmable Gate Array (FPGA) on the myRIO was setup to produce a digital waveform to control a set of complementary metal-oxide-semiconductor (CMOS) analogue switches that select between the individual microphone elements. A single analogue signal was captured at a rate of 500 kHz; 200 samples were captured for each element before switching to the next element meaning that 1000 samples were taken overall. The real-time processor chip on the myRIO performed DFTs at 39 kHz, 39.5 kHz, 40 kHz, 40.5 kHz and 41 kHz during the acquisition stage. Where null regions were detected, the process switched to using data from the centre three microphones, and DFTs were performed at 39.17 kHz, 40 kHz, and 40.83 kHz. The method described in Section 2.2 was used in LabVIEW 2015 to predict the source’s one-dimensional heading.

The transmitter was securely mounted and fixed in position, 0.5 m from the array centre and carefully aligned so that the array was illuminated in the main beam. The microphone array was mounted on a positioner and rotated around its axis as shown in Figure 1. For each bearing angle measured, 1000 samples were taken and the DFT amplitudes were analysed to estimate the source direction $\theta_{est}$ and this estimation was compared with the known direction *θ*. In addition, performance data was produced using LabVIEW’s “Tick Count” facilities.

**Figure 9:**
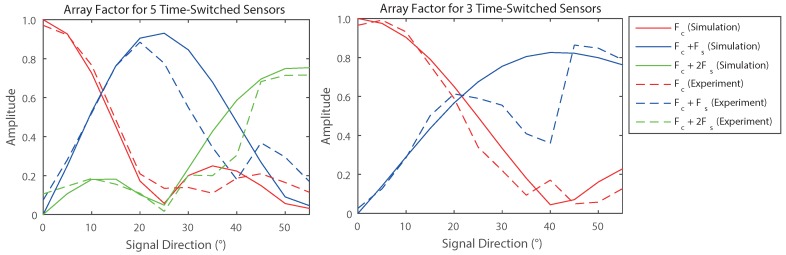
The received harmonic content of an experimental five-element linear microphone array compared to the expected numerical results.

Figure 9 shows that the beam patterns of the constructed unit are in good agreement with those predicted theoretically. It can also be seen that there are a few distortions in the patterns of the outer-harmonics; there are many possible explanations for this but the theory assumes omnidirectional receivers and does not account for the presence of a surface behind the array or the complexities of real transducers (e.g., intra-element coupling). Nonetheless, Table 1 shows that the results represent good DoA performance and agree with the overall trends that would be predicted. It is clear that the system is generally more accurate when using the data from a larger number of elements, but the use of switching to using the data from three elements has proved beneficial in the cases where the signal direction was close to one of the harmonic’s null regions ($23.6^{\circ }$).

The mean average time taken to convert the set of eight DFTs to an angle estimation was 29 μs. The time to perform the method described in [15] was also measured to compare the proposed method’s computational complexity; the mean average time taken to convert the two DFTs to an estimation in this case was 41 μs. This is expected as the handling of complex arithmetic is usually significantly slower in these systems.

## 5. Conclusions

A direction-finding method with both low hardware and computational requirements has been presented and introduced for acoustic signals. The paper has presented both numerical simulations and a full implementation of the algorithm using MEMS sensors to achieve fast direction-finding with ultrasonic frequencies; the use of small MEMS sensors makes practical implementations of ultrasonic acoustic arrays possible. The technique was demonstrated using a prototype system containing an FPGA and a real-time processor, but the method can be implemented using any single device with one analogue input and digital lines to switch between sensors in a circuit.

Furthermore, since the proposed method only requires real-valued magnitudes of the frequency content, it is possible to use optimised algorithms, such as the Goertzel algorithm [19], to compute the amplitudes of the harmonic content for the array, minimising the processing time and complexity needed to obtain a DoA estimation.

It is acknowledged that more computationally demanding estimators have been proposed and that in the case of [15], these make use of highly sensitive functions that may require careful handling of complex arithmetic. However, the technique demonstrated in this paper has obtained good DoA resolution in comparison and can be performed using relatively simple systems that can be adapted to suit a variety of requirements. If the system to be implemented requires high angular accuracy in the presence of noise, then the use of a greater number of microphones is preferable. If the system is required to find positions of signals that are short in duration, then it is possible to reduce the number of samples per microphone whilst keeping consistency in the direction-finding resolution.

## Figures and Tables

**Table 1:** The estimation results obtained from the system attempting to locate a source at various Direction of Arrivals (DoAs). The estimates marked in **bold** are the estimates chosen by the system as its solution.

*θ*	$\theta_{\mathrm{est}}$ Using 5 Elements	$\theta_{\mathrm{est}}$ Using 3 Elements	Absolute Error ($|\theta_{\mathrm{est}}-\theta |$)
00	**−2.457**	−3.034	2.457
05	**05.519**	04.714	0.519
10	**09.523**	09.815	0.477
15	**14.390**	16.608	0.610
20	**21.048**	21.310	0.924
23.6	19.853	**24.331**	0.731
25	20.094	**26.542**	1.542
30	**31.533**	29.953	1.533
35	**34.440**	34.027	0.560
40	**42.142**	28.376	2.142
45	**42.777**	39.570	2.223
50	**44.499**	39.147	5.501
55	**47.483**	36.020	7.517

## References

[1] Bouchard C., Havelock D.I., Bouchard M. (2009). Beamforming with Microphone Arrays for Directional Sources. J. Acoust. Soc. Am..

[2] Naidu P.S., Subramaniyan R. (1995). Direction of Arrival Estimation in the Presence of Distributed Noise Sources: Cumulant Based Approach. J. Acoust. Soc. Am..

[3] Liu C., Wheeler B.C., O’Brien W.D., Bilger R.C. (2000). Localization of Multiple Sound Sources with two Microphones. J. Acoust. Soc. Am..

[4] Schmidt R.O. (1986). Multiple Emitter Location and Signal Parameter Estimation. IEEE Trans. Ant. Propag..

[5] Roy R., Kailath T. (1989). ESPRIT-Estimation of Signal Parameters via Rotational Invariance Techniques. IEEE Trans. Acoust. Speech Signal Process..

[6] Webb P., Wykes C. (1996). High-Resolution Beam Forming for Ultrasonic Arrays. IEEE Trans. Robot. Autom..

[7] Munro W.S.H., Pomeroy S., Rafiq M., Williams H.R., Wybrow M.D., Wykes C. (1990). Ultrasonic Vehicle Guidance Transducer. Ultrasonics.

[8] Kunin V., Turqueti M., Saniie J., Oruklu E. (2011). Direction of Arrival Estimation and Localization Using Acoustic Sensor Arrays. J. Sens. Technol..

[9] Harput S., Bozkurt A. (2008). Ultrasonic Phased Array Device for Acoustic Imaging in Air. IEEE Sens..

[10] Shanks H.E., Bickmore R.W. (1959). Four-Dimensional Electromagnetic Radiators. Can. J. Phys..

[11] Shanks H.E. (1961). A New Technique for Electronic Scanning. IRE Trans. Ant. Propag..

[12] Li G., Yang S., Nie Z. (2010). Direction of Arrival Estimation in Time Modulated Linear Arrays With Unidirectional Phase Center Motion. IEEE Trans. Ant. Propag..

[13] Liu C., Yang S., Zhu Q. Direction of Arrival Estimation based on Time-Modulated Antenna Array. Proceedings of the 2014 IEEE Antennas and Propagation Society International Symposium (APSURSI).

[14] Tennant A. (2010). Experimental Two-Element Time-Modulated Direction Finding Array. IEEE Trans. Ant. Propag..

[15] He C., Liang X., Li Z., Geng J., Jin R. (2015). Direction Finding by Time-Modulated Array With Harmonic Characteristic Analysis. IEEE Ant. Wirel. Propag. Lett..

[16] Tong Y. (2013). Time Modulated Linear Arrays. Ph.D Thesis.

[17] Tong Y., Tennant A. (2012). A Two-Channel Time Modulated Linear Array With Adaptive Beamforming. IEEE Trans. Ant. Propag..

[18] Knowles Accoustics SPU0410LR5H-QB Product Datasheet. http://www.knowles.com/eng/content/download/5755/91802/version/3/file/SPU0410LR5H-QB+revH.PDF.

[19] Goertzel G. (1958). An Algorithm for the Evaluation of Finite Trigonometric Series. Am. Math. Mon..

